# A diploid wheat TILLING resource for wheat functional genomics

**DOI:** 10.1186/1471-2229-12-205

**Published:** 2012-11-07

**Authors:** Nidhi Rawat, Sunish K Sehgal, Anupama Joshi, Nolan Rothe, Duane L Wilson, Nathan McGraw, Praveen V Vadlani, Wanlong Li, Bikram S Gill

**Affiliations:** 1 Wheat Genetic and Genomic Resources Center, Throckmorton Hall, Kansas State University, Manhattan, KS, 66506, USA; 2 Bioprocessing and Renewable Energy Laboratory, Department of Grain Science and Industry, Kansas State University, Manhattan, KS, 66506, USA; 3 Department of Biology and Microbiology, South Dakota State University, Brookings, SD, 57007, USA; 4 Faculty of Science, Genomics and Biotechnology Section, Department of Biological Sciences, King Abdulaziz University, Jeddah, 21589, Saudi Arabia

**Keywords:** TILLING, Reverse genetics, Triticum monococcum, Mutation frequency, Waxy, Lignin

## Abstract

**Background:**

*Triticum monococcum* L., an A genome diploid einkorn wheat, was the first domesticated crop. As a diploid, it is attractive genetic model for the study of gene structure and function of wheat-specific traits. Diploid wheat is currently not amenable to reverse genetics approaches such as insertion mutagenesis and post-transcriptional gene silencing strategies. However, TILLING offers a powerful functional genetics approach for wheat gene analysis.

**Results:**

We developed a TILLING population of 1,532 M_2_ families using EMS as a mutagen. A total of 67 mutants were obtained for the four genes studied. *Waxy* gene mutation frequencies are known to be 1/17.6 - 34.4 kb DNA in polyploid wheat TILLING populations. The *T. monococcum* diploid wheat TILLING population had a mutation frequency of 1/90 kb for the same gene. Lignin biosynthesis pathway genes- *COMT1*, *HCT2*, and *4CL1* had mutation frequencies of 1/86 kb, 1/92 kb and 1/100 kb, respectively. The overall mutation frequency of the diploid wheat TILLING population was 1/92 kb.

**Conclusion:**

The mutation frequency of a diploid wheat TILLING population was found to be higher than that reported for other diploid grasses. The rate, however, is lower than tetraploid and hexaploid wheat TILLING populations because of the higher tolerance of polyploids to mutations. Unlike polyploid wheat, most mutants in diploid wheat have a phenotype amenable to forward and reverse genetic analysis and establish diploid wheat as an attractive model to study gene function in wheat. We estimate that a TILLING population of 5, 520 will be needed to get a non-sense mutation for every wheat gene of interest with 95% probability.

## Background

Common or bread wheat *Triticum aestivum* L. is an allohexaploid having three genomes A, B and D and a huge genome size of ~17 Gb [1,2]. For gene functional analysis, all the three homoeologous loci have to be individually targeted and subsequently combined to evaluate the phenotypes. This may take years. Although this is necessary for many genes of agronomic interest, it is cumbersome for other genes where the biological function has not been validated in wheat. *Triticum monococcum* is a cultivated A-genome diploid wheat and can be used to study traits, genes, and alleles as a model for bread wheat. Establishing phenotype to genotype relationships and allocating function of variant alleles in diploid wheat is considerably straightforward. As an alternative to studying gene functions in hexaploid wheat it is feasible to mine databases of model species such as rice and *Brachypodium* and apply it to *T. monococcum*. The knowledge of gene function in diploid wheat will be applicable to hexaploid wheat and, furthermore, any useful novel trait found in diploid wheat can be transferred to hexaploid wheat via established breeding procedures.

Targeting Induced Local Lesions in Genomes (TILLING) is a powerful reverse genetics approach first developed in the model plant *Arabidopsis* and fruitfly *Drosophila melanogaster*[3,4]. Subsequently it has been successfully applied to many plants including maize [5], barley [6,7], rice [8-10], sorghum [11], hexaploid and tetraploid wheat [10-15], soybean [16], oat [17], *Brassica*[18] and, tomato [19]. TILLING has become the method of choice for the functional analysis of genes, because it can be applied to a broad range of organisms and practically any gene of interest [20]. Furthermore, mutagenesis is achieved in TILLING without the addition of transgenic DNA and is stable as compared to other reverse genetics tools like insertional mutagenesis, RNA interference (RNAi) [21,22], and genome editing using ZFNs and TALENs [23,24]. TILLING also produces a broad range of alleles including nonsense, missense, splicing and, cis-regulatory, which may be used to assign functional domains of proteins [9].

TILLING in hexaploid wheat has been reported by several groups [10,12-15,25,26]. However, TILLING in diploid wheat, although desirable, has not been attempted. To exploit the distinct advantages of TILLING in diploid wheat for functional genomics studies, we developed and characterized an EMS-induced TILLING population consisting of 1,532 fertile/partially fertile M_2_ families. The utility and mutation frequency of the population was determined using genes *waxy*, *caffeic acid O-methyltransferase 1* (*COMT1*), *4-coumarate-CoA ligase 1* (*4CL1*), and *hydroxycinnamoyl-CoA:shikimate/quinate hydroxycinnamoyltransferase* (*HCT2*). The *waxy* gene was used because mutation frequencies are already established for it in hexaploid wheat [12]. *COMT1*, *4CL1*, and *HCT2* are three important genes in the lignin biosynthetic pathway of plants [27,28], and the frequency of mutation for *COMT1* is known in a sorghum TILLING population [11] for comparative mutation frequency analysis.

## Results

### Development of the *T. monococcum* TILLING population

**Figure 1:**
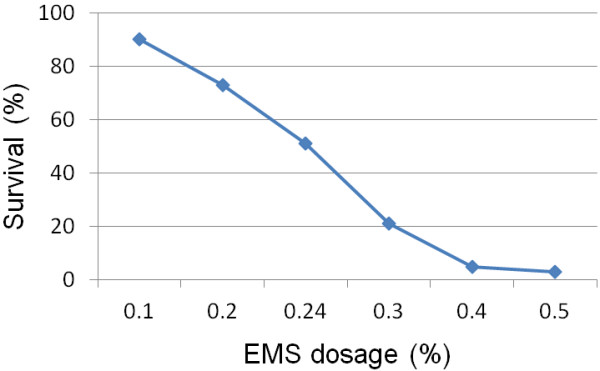
**EMS Dosage optimization curve.** A dose of 0.24 % EMS was applied to develop *T. monococcum* (TA4342-96) TILLING population at a kill rate of 51%.

Our pilot experiment showed that a 0.24 % EMS mutagenesis treatment of *T. monococcum* accession TA4342-96 had a kill rate of 50% and produced many phenotypic mutants [Figure 1]. Ninety percent of the surviving M_2_ individuals were fully/partially fertile. Increasing the dose would compromise the survival and fertility of plants, and a lower dose would not yield enough mutations to saturate the genome. Out of a total of 3,700 *T. monococcum* seeds treated (M_0_), 1,890 M_1_ plants survived and set seed. One seed per spike was sown from each M_1_ plant to reduce mutation redundancy and 1,700 viable M_2_ plants were obtained. One hundred and sixty-eight M_2_ plants were sterile and did not produce any seeds. Plant tissue was collected and catalogued from the fully/partially fertile 1,532 M_2_ plants and selfed M_3_ seed from these was archived.

**Figure 2:**
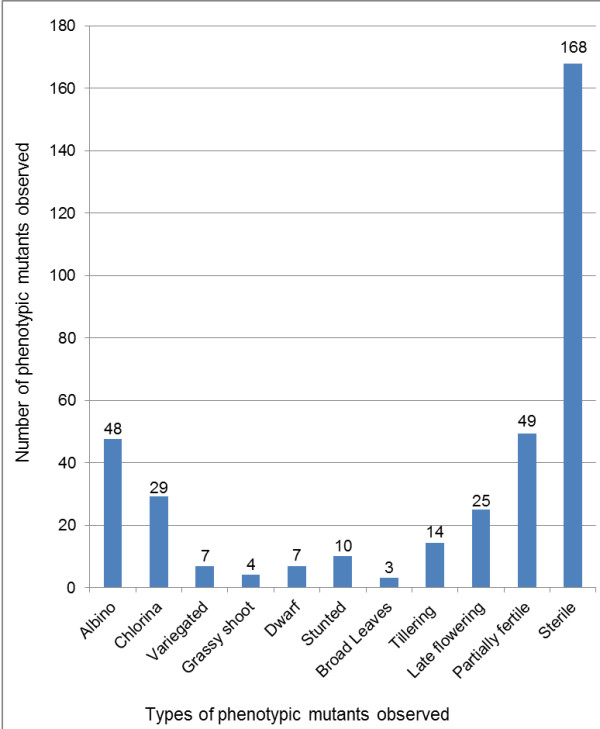
Number and types of phenotypic mutants observed in the TILLING population.

Several phenotypic mutants, such as albino, chlorina, striped, dwarf, grassy-shoot, broad leaf, early/late flowering, and delayed senescence were observed in the M_2_ generation. Figure 2 gives the number of phenotypic mutants of each type observed in the population.

### Sequencing of the *T. monococcum* lignin genes

Primers were designed to cover only the exonic regions of the three lignin genes studied, so as to avoid translationally-inactive intronic regions, using publically available hexaploid wheat mRNA sequences, rice genomic sequences, and wheat-rice homology. The exons of *T. monococcum COMT1*, *HCT2*, and *4CL1* were PCR amplified and sequenced using the designed primers. The sequences were submitted to NCBI. The Genbank accession numbers for *T. monococcum COMT1*, *HCT2*, and *4CL1* genes (exonic regions) are JX473722, JX473723, and JX473724, respectively.

### Mutant identification by TILLING

Four genes: *waxy*, *COMT1*, *4CL1*, and *HCT2* were used to characterize the mutation frequency in the TILLING population (Table 1). For screening the *waxy* locus, two primer pairs, W1 [29] and DOS (designed from sequence of *T. monococcum wx-Tma* gene, Genbank Accession No. AF110373), were used. We found a total of 27 mutants for the *waxy* locus, 13 for W1, and 14 for DOS, by scanning a total of 2,438 kb of DNA. Two primer pairs each were designed for *COMT1*, *4CL1*, and *HCT2* covering their exonic regions. For *4CL1*, however, last three exons were combined because of their very small size to generate a single amplicon (primer pair *4CL1_C*). *COMT1_A* and *COMT1_B* were designed on the basis of the hexaploid wheat *COMT1* gene sequence. For *COMT1_A* and *COMT1_B*, 6 mutants each were found in a total of 949 kb scanned. For *HCT2_A* and *HCT2_B*, 4 and 12 mutants, respectively, were detected by scanning a total of 1,452 kb. Five and seven mutants were found for *4CL1_B* and *4CL1_C*, respectively, in a total of 1,248 kb scanned*.* The mutation frequency for the *waxy* gene was found to be 1/90 kb, whereas for *COMT1*, *HCT2*, and *4CL1* the frequencies were 1/86 kb, 1/92 kb, and 1/100 kb, respectively. The overall mutation frequency of our TILLING population on the basis of the all four genes was 1/92 kb.

**Table 1:** **Details of the primers and mutation frequencies found for genes in the*T. monococcum*TILLING population**

**Primer Name**	**Gene**	**Sequence 5′-3′**	**Number of mutants identified**	**Product size**	**No. of bases screened**	**Gene-wise mutation frequency**
W1_F	*waxy*	TTGCTGCAGGTAGCCACAC	13	931	1141034	1/90 kb
W1_R		CTCAAGTGCTGCCTGGCAGAGAA				
DOS_F		AGGACAACCAGCTACGCTTCAG	14	1058	1296685	
DOS_R		TGATGACGTACCAGGAGAACGA				
COMT1_AF	*COMT1*	CATGTACGCTCTCCAGCTCGTCTC	6	379	464502	1/86 kb
COMT1_AR		AGCTCTCCATGAGGACCTTGTC				
COMT1_BF		GAACCACTCCATCATCATCACC	6	466	571130	
COMT1_BR		CGAACTCCCTCTCGTACCTCTC				
HCT2_AF	*HCT2*	CGCCGAGCGTCTACTTCTTC	4	308	377485	1/92 kb
HCT2_AR		GAGGAGCGGGTAGGAGGAGAT				
HCT2_B		GGCCTGCACTTCATCAACTC	12	877	1074851	
HCT2_BR		CGGTGCTTCTTTCTTTCTGC				
4CL1_BF	*4CL1*	AGGACGCCTTCATGGCTAAGATCC	5	317	388515	1/100 kb
4CL1_BR		TCATGATCTGCTCGCCGCGGATGC				
4CL1_CF		AGAGTCCACCAAGAACACCATC	7	701	859146	
4CL1_CR		CTGGCTCTCAAGTCCTTCCTC				

Of the total 67 mutants identified, 51 were heterozygous and 16 were homozygous, which fits the expected ratio of 2:1 heterozygous:homozygous M_2_ plants (χ^2^=1.796, P<0.01). Table 2 gives the details of the mutants found for all four genes.

**Table 2:** **Nature of mutations found in the*T. monococum*TILLING population**

**Primer set (F+R)**	**Plant ID (Pool-Plate-rowcolumn)**	**Base pair change**	**Homo/heterozygous**	**Type of mutation**	**Amino acid change**	Position of amino acid*	**SIFT score**	**PSSM score**
COMT1_A	1-4-G6	C>T	Hetero	Mis-sense	Thr>Met	90	0.14	0
	2-1-E6	G>A	Hetero	Silent	-	91	-	-
	4-1-A3	G>A	Homo	Mis-sense	Ala>Lys	95	0.36	+1
	4-1-A11	G>A	Hetero	Mis-sense	Glu>Lys	92	0.24	+1
	5-7-D6	G>A	Hetero	Mis-sense	Ala>Thr	65	0.25	+4
	6-10-G8	G>A	Homo	Mis-sense	Glu>Lys	116	0.23	+1
COMT1_B	1-2-B8	C>T	Hetero	Mis-sense	Ala>Val	275	0.45	+4
	3-3-E4	G>A	Hetero	Silent	-	298	-	-
	4-3-B10	G>A	Hetero	Mis-sense	Met>Ile	261	0.55	0
	4-4-H5	G>A	Homo	Mis-sense	Glu>Lys	271	0.41	−1
	5-8-A1	G>A	Hetero	Mis-sense	Glu>Met	298	0.16	−4
	6-10-F3	C>T	Hetero	Mis-sense	Pro>Ser	248	0.42	+2
HCT2_A	1-3-B12	G>A	Hetero	Mis-sense	Gly>Arg	131	0.25	−2
	2-5-G6	G>A	Hetero	Silent	-	100	-	-
	5-8-C3	C>T	Homo	Silent	-	72	-	-
	5-6-C11	G>A	Hetero	Silent	-	85	-	-
HCT2_B	1-1-A4	G>A	Hetero	Mis-sense	Glu>Lys	208	0.45	+1
	1-2-E7	G>A	Homo	Silent	-	371	-	-
	2-1-E12	C>T	Hetero	Silent	-	395	-	-
	2-6-H8	G>A	Hetero	Mis-sense	Asp>Asn	330	0.56	−2
	3-2-G8	C>T	Homo	Silent	-	359	-	-
	3-2-F5	C>T	Hetero	Mis-sense	Thr>Met	314	0.19	−1
	4-2-A9	G>A	Homo	Mis-sense	Ala>Thr	275	0.50	−3
	4-1-B1	C>T	Hetero	Silent	-	308	-	-
	4-3-G4	G>A	Hetero	Mis-sense	Val>Ile	310	0.59	+2
	5-8-C8	C>T	Hetero	Mis-sense	Leu>Phe	267	0.37	+2
	6-10-A8	G>A	Hetero	Silent	-	287	-	-
	6-10-F11	G>A	Hetero	Silent	-	414	-	-
4CL1_B	2-1-H5	G>A	Hetero	Mis-sense	Met>Ile	405	0.31	−3
	3-4-C10	C>T	Hetero	Mis-sense	Thr>Met	345	0.11	−3
	4-1-E2	G>A	Hetero	Silent	-	340	-	-
	5-8-A10	G>A	Homo	Mis-sense	Gly>Asp	370	0.28	−7
	6-9-G10	G>A	Hetero	Non-sense	Trp>Stop	338	-	-
4CL1_C	1-2-D3	C>T	Hetero	Intronic	-	-	-	-
	1-1-A7	G>A	Hetero	Intronic	-	-	-	-
	3-4-B7	C>T	Hetero	Intronic	-	-	-	-
	4-3-B6	C>T	Hetero	Intronic	-	-	-	-
	5-7-E8	G>A	Homo	Mis-sense	Val>Ile	476	0.27	+1
	5-6-B8	C>T	Hetero	Silent	Gly	485	-	-
	6-9-H8	C>T	Hetero	Silent	Gly	534	-	-
W1	1-2-C5	G>A	Hetero	Mis-sense	Gly>Ser	95	0.20	−4
	1-1-D7	C>T	Hetero	Mis-sense	Ala>Val	58	0.19	Not found**
	2-1-H5	C>T	Hetero	Silent	-	104	-	-
	2-5-E5	C>T	Hetero	Intronic	-	-	-	-
	3-3-B3	G>A	Homo	Mis-sense	Gly>Glu	101	0.17	−1
	3-3-E3	C>T	Hetero	Intronic	-	-	-	-
	3-2-G3	C>T	Hetero	Silent	-	60	-	-
	4-3-B5	G>A	Homo	Silent	-	51	-	-
	4-4-F9	G>A	Hetero	Intronic	-	-	-	-
	4-4-G9	G>A	Hetero	Mis-sense	Arg>His	153	0.09	+1
	5-8-E6	C>T	Homo	Intronic	-	-	-	-
	6-10-F1	C>T	Hetero	Intronic	-	-	-	-
	6-9-H7	G>A	Hetero	Mis-sense	Glu>Lys	143	0.14	−1
DOS	1-4-D8	G>A	Hetero	Silent	-	253	-	-
	1-2-G4	G>A	Hetero	Mis-sense	Glu>Lys	328	0.12	−2
	2-5-D6	G>A	Hetero	Intronic	-	-	-	-
	2-6-G4	G>A	Homo	Intronic	-	-	-	-
	3-2-D1	G>A	Homo	Silent	-	359	-	-
	3-3-G9	G>A	Hetero	Mis-sense	Val>Met	254	0.10	−6
	3-4-H2	C>T	Hetero	Mis-sense	Ala>Val	305	0.13	−3
	4-3-F4	G>A	Homo	Intronic	-	-	-	-
	4-1-H2	G>A	Hetero	Silent	-	294	-	-
	5-7-A8	C>T	Hetero	Mis-sense	Ala>Val	399	0.11	+2
	5-7-D1	G>A	Hetero	Silent	-	315	-	-
	5-5-H1	C>T	Hetero	Mis-sense	Ala>Val	252	0.10	+2
	6-10-E6	G>A	Hetero	Silent	-	277	-	-
	6-9-F6	C>T	Homo	Silent	-	346	-	-

*Position of amino acid acid change in rice protein, where wheat reference not available.

**PSSM not found, compositionally biased region, not used in domain database search.

### Sequencing of the mutants

All 67 mutations identified were G/C > A/T transitions. Of the 27 mutations identified for the *waxy* locus, eight were in the non-coding regions. Nine of the 19 mutations in the coding region were silent, because the SNP occurred in the third wobbling base of the triplet codon. The remaining 10 mutants of the *waxy* locus had mis-sense mutations. For *4CL1*, one non-sense mutation was found in 6-9-G10, where a G>A mutation converted a tryptophan to a stop codon. Of the remaining 12 mutations, four were in the introns, three were silent, and four were mis-sense. Because the exon size of the *HCT2* and *COMT1* genes encompassed the amplicon, all identified mutations were within the coding regions of these genes. In these two genes, 39% of the mutations were found to be silent; the remaining 61% were mis-sense.

To assess the probability of phenotypic effect of the mis-sense mutations, bioinformatic tools SIFT (Sorting Intolerant From Tolerant) [30] and PSSM (Position specific scoring matrix) [31] were employed. For a mutation to be intolerant, the SIFT scores should be less than 0.05, whereas high positive PSSM scores (>6) suggest deleterious effects of the mutation on protein function. The SIFT scores of all the identified mis-sense mutations in *COMT1*, *HCT2*, and *4CL1* were more than 0.1 and their PSSM scores were low, indicating the mis-sense mutations were notdeleterious. All SIFT scores were in agreement with the PSSM values.

### Lignin quantification

Wild type *T. monococcum* was found to contain 21.1% lignin content in their spikelets. Homozygous M_3_ individuals of four mutants from each *COMT1*, *4CL1*, and *HCT2* primer pair were analyzed in six replications for lignin content. The mutant with non-sense mutation in *4CL1_B* had 19.6% lignin content in the spikelets, which is 7.7% lower than that in the wild type. However, this difference was found to be statistically insignificant (χ^2^ =0.124, P<0.05). Other mutants had lignin content in the range of 17.3-22.7%, which also were not significantly lower than the wild type. This was as expected based on their SIFT and PSSM predictions.

## Discussion

TILLING, based on chemically induced mutations, has become a method of choice for reverse genetics studies, because it can be applied to a broad range of organisms to scan for mutations in any gene of interest. Greene et al. [20] showed that EMS-induced mutations are randomly distributed across the entire genome and, theoretically, all the genes are responsive to mutagenesis and TILLING, contrary to post-transcriptional gene silencing (PTGS) techniques, which have variable success rates with some organisms/genes. Once a good TILLING resource has been developed for an organism, it can be used to study any gene function in that organism, whereas with other reverse genetics study approaches, resources have to be developed individually for each gene. Additionally, allelic series of genes obtained by TILLING can provide information on important domains or amino acids within the protein of interest [9,32]. In *Arabidopsis*, hexaploid wheat, and maize, more than 99% of the EMS-induced mutations are G/C > A/T transitions [5,12,20]. All the mutations found in diploid wheat were also G/C > A/T transitions. In barley, rice, soybean and *Drosophila*, however, other transitions and transversions, have been reported to occur in 10-30% of the cases [6,9,16,33].

Wheat has different levels of polyploidy ranging from diploid to hexaploid. Polyploids, such as hexaploid bread wheat, tetraploid durum wheat, and hexaploid cultivated oat, are easily amenable to TILLING probably because genome buffering enables plants to survive a high level of mutation [12-14,17]. However diploid plants like *Arabidopsis*, rice, diploid wheat, and barley have non-redundant complements of genes and are expected to be less tolerant of increasing mutation density. Therefore, creating mutant populations in a diploid species is a very careful exercise, because the mutagenesis treatment has to be such that it induces mutation but at the same time does not lead to all defective or sterile plants [32]. Hexaploid and tetraploid wheat can tolerate an EMS treatment as high as 1% and 0.75%, respectively, whereas with our diploid wheat only 3% of the M_1_ plants survived a 0.5% EMS treatment. Similar low tolerance rates with EMS mutagenesis have been reported in other diploid plants such as *Arabidopsis*[21,34], rice [8,9], barley [6,35,36], and sorghum [11]. The *T. monococcum* TILLING population developed in this study can be used as a model for functional genomics, applicable to tetraploid and hexaploid wheat. Presence of only one homoeologous locus for any gene in diploid wheat, as against two or three homologous loci in tetraploid wheat and hexaploid wheat, respectively makes this population useful for validating gene function and structure. Information from model plants like rice, *Brachypodium*, or *Arabidopsis* can be applied directly to diploid wheat. In this study information from rice genomic database was used to predict gene structure of *T. monococcum* lignin genes and primers were designed for covering only exonic regions for mutation detection to find maximum functional mutations.

*Cel-*I-based single-nucleotide specific mismatch cleavage was used to detect mutations in the genes of interest as it is a robust technique of identifying SNPs with nearly zero false detection rate [11,13,20]. Agarose gel platforms were used due to their quick availability and cost effectiveness [25,36,37]. However, this method is not very high throughput due to the increased signal-to-noise ratio with higher pooling in agarose gels. With the reduction in costs of next generation sequencing (NGS), it is now possible to TILL several genes simultaneously using TILLING-by-sequencing [10,38,39]. Screening for mutations using NGS can be done along two lines; first by sequencing pooled amplicons [10,38] and second by sequence capture [39,40]. The former has high throughput for the number of individuals for some genes, whereas the latter is suitable for scanning many genes within a few individuals [10]. Both approaches involve a considerable amount of work preparing sequencing libraries. Analysis of NGS reads also requires extensive software treatment and, because the scope of our investigation was limited to a few genes, it was more practical to use the traditional *Cel*-I assay.

The *waxy* gene, encoding granule bound starch synthase I (GBSSI), has been widely studied in wheat. Knockouts of *waxy* have starch composed entirely of amylopectin and no amylose, and have been developed by combining null alleles at all the three loci (ABD) by breeding [41]. TILLING has been used to identify mutants at all the functional *waxy* homoeologous loci in hexaploid wheat [12,25]. Slade et al. [12] reported a very high mutation frequency of 1/18 kb DNA screened in hexaploid bread wheat, followed by that of tetraploid durum wheat (1/34 kb DNA). The low mutation frequency for the same *waxy* locus in our diploid wheat TILLING population (1/90 kb) indicates that a decrease in ploidy level reduces tolerance to mutations due to lack of genome buffering.

The *4CL* genes are a multigene family and have been investigated extensively in dicots and gymnosperms for their role in lignin biosynthesis [42-45]. In dicots such as *Arabidopsis* and *Populus trichocarpa*, *4CL* genes have been classified into two types, type I (*4CL1*, *4CL2*, and *4CL4*), regulate lignin accumulation, and type II (*4CL3*) is mainly responsible for the synthesis of other phenolic compounds [42,46]. However, monocots have different monolignol composition and phenolic compounds and alternative genes have been proposed to play varying roles in lignin synthesis [47,48].

Gui et al. [48] studied the catalytic properties and roles of all the five rice *4CL* genes in lignin biosynthesis by suppressing their expression using antisense RNA. Suppression of only *4CL3* resulted in a significant decrease in lignin content, poor plant growth, and abnormal anther development. Suppression of *4CL1* did not affect lignin content in rice. Thus, it was concluded that monocots have different genes than dicots playing distinct roles in the lignin biosynthesis pathway [48]. Our findings with a truncation mutant of the wheat *4CL1* gene showing insignificant reduction in lignin content may also be due to the same reason. Our previous RNAi studies to suppress *4CL1* genes in hexaploid wheat and did not detect any change in lignin content (Bi et al. unpublished results). More experimental proof is needed however, before ruling out *4CL1* as the major determinant in monocots in general. Based on sequence similarity, another class, type III *4CL*, was added in monocots by Gui et al. [48] (including rice *4CL3*, *4CL4*, *4CL1* and *4CL5*) to the known dicot classes.

For the *COMT1* gene, Xin et al. [11] screened 768 EMS mutagenized lines of sorghum by TILLING and found two mutants by screening 624.4 kb. The frequency of mutation in the gene was 1/312 kb in their population. Our TILLING population had quite a high mutation frequency for the *COMT1* gene (1/92 kb). However, brown midrib was not observed in any of the mutants because none of the identified mutants for *COMT1* had a truncation or mis-sense mutation with a deleterious effect.

Diploid species such as barley, sorghum, pea, peanut, and rice have low mutation frequencies ranging from 1/300 to 1,000 kb compared to the polyploids such as oat, bread wheat, and soybean [5,6,10,11,15,16]. Our TILLING population has higher frequency of mutation (1/104 kb) than the other diploid grasses (Table 1). Martin et al. [34] developed a TILLING population of *Arabidopsis* in the Landsberg *erecta* genetic background and were able to achieve a high mutation frequency (1/89 kb) by selecting for plants with low fertility in the M_1_ generation. The frequencies of mutations vary across genes, species, mutagen, treatment procedure, and detection strategies [13]. In fact, in wheat, different frequencies of mutations have been reported for different genes, sometimes even in the same genetic background [13,14]. Frequencies of mutation for the same gene and genotype are variable even across the three wheat genomes [12,13]. Variable frequencies of mutation have been reported in wheat for *Starch branching enzyme IIa* (*SBEIIa,* 1/49 to 1/124 kb), *Wheat Kinase Start1* (*WKS1*, 1/37 to 1/60 kb), *WKS2* (1/36 to 1/42 kb), *waxy* (1/12 kb to 1/41 kb), *Puroindoline a* (*Pina*, 1/31 kb), and *Pinb* (1/29 kb) [12,25,26]. The mutation frequency (1/92 kb) of our TILLING population establishes it as a suitable high-throughput, reverse genetics resource of wheat.

About 5% of the mutants found in TILLING populations of *Arabidopsis*, rice, and wheat have been reported to be truncation mutants [12,32,34,49]. Because diploids have a lower frequency of mutation, larger populations are needed to get at least one truncation event per gene compared to polyploids. Table 3 summarizes the frequencies of mutations reported in various species and gives an estimate of the approximate number of lines required to get at least one truncation mutant with a 95% probability for each gene in a typical 1kb coding region screened, assuming 5% of the total mutations are truncations. With the current mutation frequency in our *T. monococcum* population, about 5,520 mutagenized individuals will be needed to get one stop codon for any gene of interest with 95% probability.

**Table 3:** Estimated population size required to identify a truncation mutant in some TILLING populations* (Modified from Parry et al. 2009 [32])

**Species**	**Ploidy**	**Frequency of mutation**	**Population size used**	**Population size required**	**Reference**
*Arabidopsis*	diploid	1/300 kb	3 072	18 000	Greene et al. 2003 [20]
	diploid	1/89 kb	3 712	5 340	Martin et al. 2009 [34]
Sorghum	diploid	1/526 kb	768	31 560	Xin et al. 2008 [11]
Rice	diploid	1/294 kb	768	17 640	Till et al. 2006 [51]
	diploid	1/135 kb	767	8 100	Suzuki et al. 2008 [49]
Barley	diploid	1/1000 kb	9 216	60 000	Caldwell et al., 2004 [6]
	diploid	1/374 kb	4 906	22 440	Talame et al. 2008 [7]
Einkorn wheat	diploid	1/92 kb	1 532	5 520	This study
Durum wheat	tetraploid	1/40 kb	768	2 400	Slade et al., 2005 [12]
	tetraploid	1/51 kb^a^	1 386	3 060	Uauy et al., 2009 [13]
*Arabidopsis*	tetraploid	<1/100 kb	528	6 000	UC Davis Genome Center
Bread wheat	hexaploid	1/24 kb	1 152	1 440	Slade et al., 2005 [12]
	hexaploid	1/23-38 kb^b^	2 348	1 380	Dong et al., 2009 [25]
	hexaploid	1/38 kb^a^	1 536	2 280	Uauy et al., 2009 [13]
Oat	hexaploid	1/20-40 kb	2 550	1 200	Chawade et al. 2010 [17]

*at 95 % probability in some TILLING populations, assuming 5% of the mutations being non-sense.

^a^normalized to 50% G/C content.

^b^depending upon cultivar and EMS treatment.

## Conclusions

The TILLING population developed in diploid wheat will be a useful genetic resource as a model system for studying wheat gene function as a complement to similar analysis in polyploid wheat. Because a population of at least 5,520 M_2_ individuals will be needed to obtain at least one knock-out per gene, a new set of mutagenized population in the same genetic background is currently being developed to increase the population size. Seed of this population are being increased and will be made available after the M_4_ generation for interested workers upon request. Next generation sequencing will be utilized to characterize mutation in genes of interest and increase the high throughput of this resource for wheat functional genomics analysis.

## Methods

### Plant material and EMS mutagenesis

Diploid wheat, *Triticum monococcum* subsp. *monococcum* (accession number TA4342-96) was used to develop the TILLING population. TA4342-96 is a spring-type genotype and has a planting to heading date of ~90 days. All the plants were grown in greenhouse at 20-25°C with a light period of 16 h.

To determine the appropriate concentration of the mutagen EMS, two rounds of tests were made. First, the dose of the EMS needed to achieve 40–60 % survival among the M_1_ plants was determined. Five sets of 50 seeds of TA4342-96 were soaked in water in 250ml glass flasks for 8 hours of imbibition on a shaker at 75 rpm and then treated with five different doses (0.1, 0.2, 0.3, 0.4, and 0.5 %) of EMS for 16 hours on shaker at 75 rpm. The treated seeds were washed under running water for 8 hours and then transplanted individually into root trainers. Observations were made 15 days after transplanting to estimate the survival frequency. EMS doses 0.2 and 0.25% were nearest to the targeted percent survival of plants. A second round of experiment was performed with six doses of EMS (0.20, 0.21, 0.22, 0.23, 0.24, and 0.25%) using 100 seeds per treatment. A kill rate of 50% was assumed to be desirable, because it generated several phenotypic mutants and at the same time yielded majority of the surviving plants fully/partially fertile. The 0.24% EMS treatment gave the desirable kill rate near 50% and was used as the dose of choice for treating a total of 3,700 *T. monococcum* seeds (Figure 1). The M_1_ population (derived from the M_0_ EMS-treated seed) was selfed. A single M_2_ was grown from every M_1_ plant to prevent genetic redundancy. From a total 1,700 M_2_ individuals 168 were sterile. Tissue was collected, and the seed cataloged at maturity for all 1,532 fertile/partially fertile M_2_ individuals of the TILLING population.

### Development of DNA pools

Leaf tissue from all 1,532 M_2_ individuals was collected at the four-leaf stage in 96-well blocks. DNA was isolated using a Qiagen Biosprint 96 robot with Biosprint 96 DNA plant kit (Qiagen, Valencia, CA) according to the manufacturer’s instructions. DNA was quantified on a Nano-drop and normalized to 25ng/μl in 96-well blocks. Subsequently, 4x pooling was done using 200μl of normalized DNA from each pool member. The mutants were catalogued and their DNA was identified with a unique ID as Pool-Plate-Row-Column.

### Design of primers for genes of interest

Four genes, *waxy*, *COMT1*, *4CL1*, and *HCT2*, were used to characterize the mutation frequency of the TILLING population.The *waxy* locus, with a known frequency of mutation in hexaploid and tetraploid wheat [12,25], was scanned for making a direct comparison of mutation frequency with ploidy level. However, the A-genome specific primers of Slade et al. [12] (WXA2 and WXA3) did not amplify in our *T. monococcum* accession. The W1 primer pair from Yan et al. [29] was used, and an additional primer pair, named DOS, was designed from *T. monococcum wx-Tma* gene (Genbank Accession No. AF110373). Both these primer pairs covered the exonic as well as the intronic regions of the gene. The primer pairs W1 and DOS produced amplicons of 931 and 1,058 bp, respectively, and were designed such that they had an overlap of 47 bp.

Three important genes of the lignin biosynthetic pathway, *COMT1*, *HCT2*, and *4CL1*, were also used to validate the frequency of mutation found at the waxy locus. Complete cds of the hexaploid wheat *COMT1* gene (Genebank Accession no. AY226581) was used to design primer pairs. Two primer pairs, *COMT1_A* and *COMT1_B*, were designed to cover the exonic regions on the basis of the hexaploid wheat *COMT1* gene sequence (Genebank Accession no. AY226581). For genes *4CL1* and *HCT2*, the TaGI database (version 11.0, http://compbio.dfci.harvard.edu/cgi-bin/tgi/gimain.pl?gudb=wheat) was searched by BLAST against rice loci LOC_Os06g44620 and LOC_Os02g39850, respectively. TC284202 and TC304595 were used to design two primer pairs each for *4CL1* and *HCT2*, respectively. Because full-length gene sequences were unavailable for the target genes, homology between rice and wheat genes was used to identify exons from the available wheat cDNA sequences using the web tool SPIDEY [50]. With this approach, the exonic regions of genes were determined and primers were designed to cover them. For *4CL1_C* however, the last three exons were combined to generate a single amplicon because of their very small size. Two primer pairs each, for all the three genes, covering all the exons and yielding single bright amplicons, were employed for scanning the TILLING population. The sequences of exons and encompassed intronic regions of genes *COMT1*, *HCT2*, and *4CL1* were submitted to NCBI and have been assigned Genbank accession numbers JX473722, JX473723, and JX473724, respectively.

### PCR, *Cel-*I digestion, and mutant visualization

The target regions were amplified from pooled DNA using Biolase PCR kits (Bioline, Tauton, MA, USA) in 25μl reaction volume, on a BioRad thermocycler (BioRad, Hercules, CA, USA). All 1,532 pooled M_2_ individuals were screened for mutations in all the four genes studied. A touchdown profile (95°C-5^′^, 7 cycles of 95°C-1^′^, 67-60°C-1^′^ with a decrease of 1°C per cycle, 72°C-1^′^, followed by 30 cycles of 95°C-30s, 60°C-30s, 72°C-45s, and a final extension of 72°C-7^′^) was used. PCR products were subsequently denatured and slowly reannealed to form heteroduplexes between mismatched DNA (95°C-2^′^, 5 cycles of 95°C-01s, 95-85°C-1^′^ with a decrease of 2°C per cycle, and 60 cycles of 85-25°C- 10s. Home-made *Cel*-I endonuclease was extracted from celery according to Till et al. [51] and optimized using a SURVEYOR Mutation Detection Kit (Cat. No. 706020, Transgenomic Inc., Omaha, NE, USA). For optimization, 1 μl each of *Cel*-I and enhancer from the kit, was added to 25 μl of heteroduplexed PCR product with the DNA and primers supplied with the kit. The intensity of cleaved bands was used to standardize optimum volumes of home-made *Cel*-I required to digest mismatches in the products. Two μl of *Cel*-I was added to the heteroduplexed products and incubated at 45°C for 45'. Reactions were stopped using 2.5 μl 0.5M EDTA.

**Figure 3:**
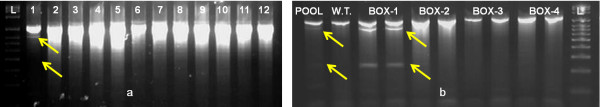
**Mutant identification in 4x pools and subsequent deconvolution.** (**A**) Identification of a mutant pool in lane 1. (**B**) Deconvolution to identify a mutant individual. In the de-pooling for each plate the first lane has M_2_ plant DNA and second lane has M_2_+wild type DNA. Plant in Box-1 is heterozygous for mutation.

The digested products were visualized on 2% agarose gels. Mutants could be identified as those products that showed cleaved bands in addition to the full-length, uncleaved product (Figure 3). The total number of bases scanned was calculated by subtracting 20% of the product size, to take into account the primer base pairs and terminal regions that escape detection. Such factors have been taken into consideration while calculating total coverage in LICOR, PAGE, and agarose gels by other workers [12,23,25].

### Deconvolution and sequencing of mutants

Pools showing mutation were deconvoluted with a similar procedure as above to identify the particular plants with the mutation. Each individual member from the mutant pool was subjected to two reactions, one of which had only the M_2_ DNA, whereas the second reaction had wild type (WT) *T. monococcum* and M_2_ plant DNA. This was done to identify the homo/heterozygosity of the mutant individual. The mutant individual was then sequenced on an ABI3730xl (Applied Biosystems, Foster City, CA) using the manufacturer’s instructions.

Zygosity was determined by following the basic TILLING procedure in the progeny of M_2_ plants. Heterozygous M_2_ plants selected for *HCT2_A*, *HCT2_B*, *COMT1_A*, and *COMT1_B* segregated in 3:1 heterozygous: homozygous ratio in M_3_ generation. Homozygous M_2_ mutants gave all homozygous M_3_ progeny for the mutation.

### Lignin estimation

Ten seeds were grown from all the identified mutants and homozygous M_3_ individuals were isolated to estimate lignin content. Four mutants each for all the primer pairs of genes *COMT1*, *4CL1*, and *HCT2*, along with WT *T. monococcum*. The spikes of homozygous M_3_ individuals were collected upon maturity and analyzed for lignin content. Lignin content was estimated by an acetyl bromide method as described in Chawade et al. [17] with 50 mg of dry spikelet tissues in six replications for each sample. This method is sensitive for even small sample size, and is relatively easy. The least interference from non-lignin products has been reported in this procedure, because it allows complete dissolution of lignin in plant tissue and provides precise absorbance values for total lignin content [17,52].

### Calculation for the estimated population size

Assuming 5% of the mutations in a TILLING population to be non-sense, a population size yielding one non-sense for each gene of 1kb length was calculated. Based on the Poisson distribution, at 95% confidence of finding at least one non-sense mutation in any gene of 1 kb, the required population size should be three times the size of this population [Table 3].

## Abbreviations

TILLING: Targeting Induced Local Lesions in Genome; COMT1: Caffeic acid O-methyltransferase 1; 4CL1: 4-coumarate-CoA ligase 1; HCT2: Hydroxycinnamoyl-CoA:shikimate/quinate hydroxycinnamoyltransferase 2; EMS: Ethyl methane sulphonate; SIFT: Sorting Intolerant From Tolerant; PSSM: Position specific scoring matrix.

## Competing interests

Authors declare that they have no competing interests.

## Authors’ contributions

Nidhi R performed TILLING experiments, did sequence analysis, and drafted the manuscript. SKS and DLW developed the mutant population. SKS and WL helped in designing primers. DLW collected and archived the tissue and seeds stocks. Nidhi R and AJ did DNA extraction, pooling, PCRs, and *Cel*-I extraction. Nidhi R and SKS did sequencing. NR did phenotype evaluation. NMG and PV did lignin estimation of the mutants. BSG planned and headed the development of mutant population and oversaw TILLING experiments. Nidhi R and BSG were primarily responsible for drafting and revising the manuscript with contributions from co-authors. All authors read and approved the final manuscript.
